# *Cryptosporidium parvum* Infection Depletes Butyrate Producer Bacteria in Goat Kid Microbiome

**DOI:** 10.3389/fmicb.2020.548737

**Published:** 2020-10-16

**Authors:** Mohamed Mammeri, Dasiel Alvarez Obregón, Aurélie Chevillot, Bruno Polack, Christine Julien, Thomas Pollet, Alejandro Cabezas-Cruz, Karim Tarik Adjou

**Affiliations:** ^1^UMR BIPAR, Ecole Nationale Vétérinaire d’Alfort, ANSES, INRAE, Université Paris-Est, Maisons-Alfort, France; ^2^Phileo by Lesaffre, Marcq-en-Barœul, France; ^3^Centre for Nuclear Energy in Agriculture, University of Sao Paulo, Piracicaba, Brazil; ^4^School of Environmental Sciences, University of Guelph, Guelph, ON, Canada; ^5^UMR ASTRE, INRAE, CIRAD, Université Montpellier, Montpellier, France

**Keywords:** diarrhea, gut microbiome, 16S, functional traits, dysbiosis, resilience 3

## Abstract

*Cryptosporidium parvum* is an important apicomplexan parasite infecting ruminants and humans. We characterized the impact of *C. parvum* infection on the goat kid microbiome. *C. parvum* was orally administered to parasite-naïve goats, and infection was monitored for 26 days in fecal samples using immunofluorescence assay and qPCR tests. Age-matched goats served as uninfected controls. A reduction in body weight gain, diarrhea, and dehydration were observed in infected goats compared to the uninfected controls. Infection decreased the bacterial diversity 5 days post-infection (dpi), but this parameter recovered at 15 dpi. The infection altered the relative abundance of several taxa. A total of 38 taxa displayed significant differences in abundance between control and infected goats at both 5 and 15 dpi. Co-occurrence network analysis revealed that the infection resulted in a differential pattern of taxa interactions and that *C. parvum* infection increased the relative abundance of specific taxa. The 16S data set was used for metagenome predictions using the software package PICRUSt2. As many as 34 and 40 MetaCyc pathways (from 387 total) were significantly affected by the infection at 5 and 15 dpi, respectively. Notably, *C. parvum* decreased the abundance of butyrate-producing pathways in bacteria. Low levels of butyrate may increase mucosal inflammation and tissue repair. Our results suggest that the gut inflammation induced by *C. parvum* infection is associated with the reduction of butyrate-producing bacteria. This insight could be the basis for the development of novel control strategies to improve animal health.

## Introduction

*Cryptosporidium parvum* is a protozoan parasite with a worldwide distribution, capable of infecting a wide range of vertebrate hosts, including both humans and ruminants (Rahman et al., 2017). Cryptosporidiosis, caused by *C. parvum*, is a common infection of neonatal ruminants that causes high morbidity and mortality and is associated with significant economic losses. Four- to Fifteen-day-old goat kids are very susceptible to cryptosporidiosis. The clinical presentation of the disease includes watery yellow diarrhea, weight loss, and poor growth that can cause death in the infected animals (Foreyt, 1990; Noordeen et al., 2012; Giadinis et al., 2015). The neonatal period is also critical for the establishment and the development of animal microbiota (Wang et al., 2018). The gut microbiota is a complex community of microorganisms, including at least several hundred bacterial species (Wang et al., 2018). The gut microbiota constitutes a symbiotic organ that influences host physiology and the progression of parasitic diseases (Leung et al., 2018). Infection with diarrhea-producing enteropathogens disturbs microbiota composition (O’Hara and Shanahan, 2006), and microbiota modulation can be associated with either resistance or susceptibility to infection (O’Hara and Shanahan, 2006; Filyk and Osborne, 2016). Therefore, considering the gut microbiota when studying enteropathogen infections could provide new insights into the pathogenicity of parasitic diseases such as cryptosporidiosis (O’Hara and Shanahan, 2006; Filyk and Osborne, 2016).

Currently, microbiota–protozoan interactions in the gut of infected animals are poorly understood (Berrilli et al., 2012; Burgess et al., 2017). Early studies, however, suggested that susceptibility to cryptosporidial infection is influenced by the composition of the host microbiota (Harp et al., 1988). Germ-free mice that lacked a healthy microbiome were more susceptible to *C. parvum* infection than mice with a normal intestinal microbiota (Harp et al., 1988). Host microbiota composition also influenced horse susceptibility to gastrointestinal strongyles (Clark et al., 2018). More recently, the modulatory effect of *C. parvum* infection on the gut microbiota was reported in immune-compromised (Ras et al., 2015) and newborn (Mammeri et al., 2019) mouse models. Similar microbiota-modulating effects were observed with the *Strongyloides venezuelensis* infection progress in a mouse model (Afrin et al., 2019), or *Haemonchus contortus* in goats (Li et al., 2016). Some limitations of these studies are (i) the focus on the general composition of the gut microbiota without understanding the possible interactions between microbiota bacteria and (ii) the lack of functional insights into parasite–microbiota interactions. Functional metagenomics complements taxonomic studies and provides further insights into the functional structure of the microbiome in response to parasite infection (Garmendia et al., 2012). For example, as many as eight Kyoto Encyclopedia of Genes and Genomes (KEGG) functions were significantly affected by *H. contortus* infection in goats (Li et al., 2016). Based on this evidence, we hypothesized that *Cryptosporidium* infection in goats has an impact on the taxonomic and the functional profiles of the gastrointestinal bacterial and archaeal microbiome. Since very few studies on *Cryptosporidium*–microbiota interactions exist, the objective of this study was to characterize the impact of *C. parvum* infection on the taxonomic composition and the functional traits of goat kid microbiome.

## Materials and Methods

### Animals

Twenty-one-day-old male French Alpine goat kids (*N* = 20) were randomly selected for this study. Only males were included because, unlike females, their feces do not mix with the urine, allowing an unbiased assessment of fecal consistency and parasitic burden. Newborn kids were selected from a breeding group without a history of cryptosporidiosis or other health problems. In addition, before giving birth, all goat mothers were tested for the presence of *Cryptosporidium* oocysts in feces using an immunofluorescence assay (IFA) and real-time quantitative PCR (qPCR). Immediately after birth, the goat kids were separated from their mothers and were fed three times with colostrum heated for 1 h at 56°C to prevent caprine arthritis encephalitis virus infection. Then, the animals were transported to the *Plate-Forme d’Infectiologie Expérimentale* (PFIE) facility. The goat kids were randomly assigned to two groups (uninfected and infected, *n* = 10 per group). The animals in the two groups were placed in separate pens within the same room. A commercial, non-medicated milk replacement (Nectagneau, 150 g dissolved in 850 ml of water, Sermix, Languidic, France) was used to feed the animals *ad libitum* for the duration of the experiment.

### Experimental Infection

The infected group was orally inoculated with 10^6^ *C. parvum* Iowa strain oocysts (Waterborne^TM^, Inc., New Orleans, LA, United States) diluted in 5 ml sterile water following a previously described method (Viel et al., 2007). The same volume of diluent (5 ml distilled water) was administered to the uninfected group. At the end of the experiment (26 days post-infection), the goat kids were euthanized by an intravenous injection of a lethal dose of pentobarbital (10 ml per animal) following an intramuscular injection of zoletil^®^50 (20 mg/kg, IM).

### Clinical Investigation

Core body temperatures were continuously recorded throughout the experiment with an Anipill capsule^®^ (BodyCap, Hérouville Saint-Clair, France), which is a non-invasive measurement method (Meyer et al., 2017). After arriving at the PFIE facility, the animals received the capsule *via* a syringe at the back of the mouth. Heads were maintained in an extended position to help the animals ingest the capsule. The collected data were automatically transmitted by telemetry every hour. The animals were housed per group. This is a mandatory condition in this species (Directive 2010/63/UE). Weight and mortality rates were recorded daily. In addition, clinical signs of pain and distress were recorded as endpoints. The kids were euthanized when they were comatose or in very poor general health.

Fecal consistency was scored on a scale from 0 to 4, as previously described (Koudela and Jiří, 1997) with modifications. Specifically, we used four scores (0: normal saddle, without mucus; 1: pasty and thick, molded or not; 2: creamy; 3: semi-fluid; and 4: liquid) instead of three (Koudela and Jiří, 1997). The hydration score was also recorded as previously described for other ruminants (Stebbins et al., 2018) (1: normal, 2: mildly dehydrated, and 3: severely dehydrated). Fecal consistency and dehydration score averages were calculated daily.

### Sample Collection

In order not to collect feces directly from the rectum (invasive method) nor to collect feces from the floor, the animals were daily fitted with a disposable plastic bag (Ahamed et al., 2015), clearly identified with group/animal/day, allowing the feces to be stored for a maximum of 1 h before their collection in sterile boxes. This method prevents the reinfection of kids by oocysts excreted in the feces from other animals. Feces samples (250 and 200 mg) were stored at 4 and −80°C to evaluate parasite load and for DNA extraction, respectively (Mammeri et al., 2018).

### Parasite Load Quantification by IFA and qPCR

The daily shedding of *C. parvum* oocysts was measured by IFA using the commercial Merifluor *Cryptosporidium*/*Giardia* immunofluorescence assay (Meridian Diagnostics, Inc., Milano, Italy) as previously described (Mammeri et al., 2018).

Genomic DNA was extracted using a QIAmp DNA Mini Kit (Qiagen, France) according to the manufacturer’s instructions. Briefly, the samples were suspended in lysis buffer, and the oocysts were ruptured by subjecting them to an additional initial step of 10 freeze–thaw cycles (freezing in liquid nitrogen for 1 min and heating in water bath at 90°C for 1 min) (Sahraoui et al., 2019) before DNA extraction. The DNA was stored at −20°C until its use in the molecular analysis. The *COWP* gene was amplified by qPCR using a previously described protocol (Guy et al., 2003). The qPCR method was also used to quantify the parasite load in goats at 0 days post-infection (dpi), from 2 to 13 dpi, and at 15 and 20 dpi.

The parasite load was quantified by calculating the number of *Cryptosporidium* oocysts per gram of feces (OPG), which was obtained by multiplying the total number of oocysts by the dilution factor.

### DNA Extraction and Next-Generation Sequencing

For high-throughput sequencing purposes, microbial DNA was extracted using the QIAamp DNA Stool Kit (Qiagen, Hilden, Germany) with an improved protocol described in the International Human Microbiota Standard project^1^ (Angebault et al., 2018). Amplicon sequencing of the V4 variable region of the bacterial 16S rRNA gene was performed using barcoded universal primers (515F/806R) and a standardized amplicon-library preparation protocol (Metabiote^®^, Genoscreen, Lille, France)^2^ as previously described (Burdet et al., 2018). Sequencing was performed by Genoscreen^3^, where the MiSeq Illumina 2 × 250 bp chemistry (Illumina, San Diego, CA, United States) was used.

### Bioinformatic Analysis of Amplicon Sequences

The demultiplexed fastq files were pre-processed and analyzed using the QIIME2 pipeline v. 2019.7 (Bolyen et al., 2019). Briefly, the DADA2 software package (Callahan et al., 2016) was used for cleaning and correcting the fastq files, including removal of chimeras, and merging of mate reads. Taxonomic identification of amplicon sequences variants (ASVs) was performed with the QIIME2 q2-feature-classifier plugin (Bokulich et al., 2018b) trained (99%) on the 16S rRNA (full length) SILVA database (Yarza et al., 2014) (release 132). The QIIME2 taxa bar plot command and EMPeror options (Vázquez-Baeza et al., 2013) were used to view sample taxonomic profiles.

In addition, the 16S ASVs data sets were used to predict metagenome functional content in each microbiome. The metagenome predictions were performed with the bioinformatics software package PICRUSt2 (Douglas et al., 2020). Briefly, the 16S ASVs were aligned (NSTI cutoff value of 2) to a reference phylogenetic tree containing more than 20,000 16S sequence variants from prokaryotes; from here, the software predicted functional gene families and copy numbers for each specific ASV. During the process, the number of ASVs was corrected for their 16S copy number in the corresponding bacteria. Predictions were based on several gene family catalogs [i.e., Kyoto Encyclopedia of Genes and Genomes (KEGG) (Kanehisa and Goto, 2000) and Clusters of Orthologous Genes (COGs) (Sayers et al., 2009)]. As output, we obtained an enzyme profile [Enzyme Commission (EC) code and abundance] and a pathway profile, both based on pathway mapping of the MetaCyc database (Caspi et al., 2018) and also the taxa’s contribution to each enzyme and pathway.

### Statistical Data Analysis

The clinical data were expressed as mean ± standard deviation. Mortality rates were compared using Mantel–Cox χ^2^-based tests. Average daily weight gain (ADG) was explored by a general linear mixed-effects model (GLMM) to determine how ADG was influenced by *C. parvum* infection (infected and uninfected), the infection time (from 1 to 25 dpi), and their interactions (infection × time) as fixed effects. The model included the influences of each animal as random effects (i.e., because of individual genetic and physiological factors). Statistical significance was considered to be reached when *P* < 0.05. Analysis and graphs were performed using GraphPad Prism software v.8.0.1 (GraphPad Software Inc., United States).

Analyses of microbial diversity were performed, taking into account the rarity of ASV. A comparison of Faith’s phylogenetic diversity index (Faith, 2015) (α-diversity metric) among groups was performed using Kruskal–Wallis test. β-diversity was assessed by weighted UniFrac distance (Lozupone et al., 2011), and multiple comparisons were made using the permutational multivariate analysis of variance (ADONIS) test with 999 permutations. Longitudinal analyses, including comparisons of first differences (i.e., species richness in an individual at two separate time points) and first distances (i.e., degree of dissimilarity between an individual’s microbiota composition at two separate time points), were used to explore an individual’s rate of change between time points, which was then compared between uninfected and infected animals. The analyses were performed using the Qiime2 plugin q2-longitudinal algorithm (Bokulich et al., 2018a).

For the differential analysis of taxa and pathways, all reads were used (no rarefaction). To avoid bias on computing changes in abundance from compositional data, we used centered log ratio transformation of feature tables prior to the statistical tests (Gloor et al., 2017; Morton et al., 2019). Samples from all groups were compared (for taxonomic and pathway profiles) with the Gneiss test, which explore differential features and groups of features through balances (ratios of feature) (Morton et al., 2017). Furthermore, differentially abundant taxa (and pathways) were detected by comparing the logarithmic fold-change between infected and uninfected animals at 0, 5, and 15 dpi, using a generalized linear model (GLM) as implemented in the DESeq2 R package (Love et al., 2014). DESeq2 implementation includes the regularized logarithm transformation (rlog) of compositional data and a shrinkage estimation of dispersions and fold-changes of each feature, resulting in highly accurate estimates. For the Deseq2 pipeline, *p*-values were calculated using Wald test and adjusted according to the Benjamini–Hochberg false discovery rate method to avoid artificial inflation of type-I error (Benjamini and Hochberg, 1995).

The taxonomic profiles from infected and uninfected animals were used to build taxa co-occurrence networks (Friedman and Alm, 2012). The infection level (qPCR quantification) of *C. parvum* was included as a variable for constructing co-occurrence networks from infected goat kids. The SparCC method (Friedman and Alm, 2012), implemented as an R package, was used to analyze correlations among the bacterial genera, including *C. parvum*. Only significant (*P* < 0.01) and strongly positive (SparCC > 0.5) correlations were considered. Several indexes describe the topology and the strength of the networks (i.e., the number of nodes and edges, weighted degree, network diameter, modularity). The majority of calculations were performed using Gephi 0.9.2 software (Bastian and Jacomy, 2009).

### Availability of Data and Material

The data sets analyzed during the current study are publicly available at the Sequence Read Archive^4^, under the following BioProject accession number: PRJNA603642.

## Results

### A Clinical Model of Cryptosporidiosis in Goat Kids

**FIGURE 1:**
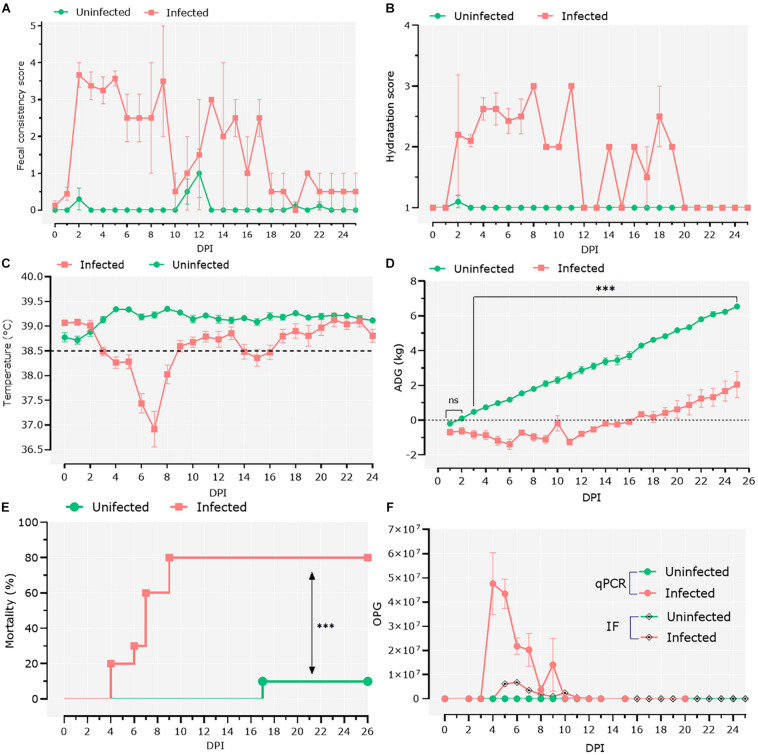
Clinical follow-up of animals. **(A)** Daily average fecal consistency score. The fecal consistency score was recorded from 0 to 4 as either: normal saddle, without mucus (0), pasty and thick, molded or not, glairy (1), creamy (2), semi-fluid (3), and liquid (4). **(B)** Daily average dehydration scores were recorded as normal (1), mildly dehydrated (2), and severely dehydrated (3). **(C)** Daily average central temperature. The orange line indicates the limit from which we consider animals in hypothermia. **(D)** Average daily weight gain (ADG). Data of ADG were explored using general linear mixed-effects model, including infection, time, and their interaction as fixed effects. The significant effect of the time–infection interaction is indicated (ns, non-significant; ^∗∗∗^*p* < 0.001). **(E)** Mortality rates were compared using Mantel–Cox χ^2^-based test; ^∗∗∗^*P* < 0.001. **(F)** Parasitic load was detected by direct immunofluorescence assay and quantitative PCR. The parasitic load was expressed as the mean of oocysts per gram of feces ± standard deviation.

To study the interactions between *Cryptosporidium* and host microbiota, we developed a model of *C. parvum* infection in goat kids (*n* = 20). Ten 1-day-old male goat kids (infected group; *n* = 10) were orally infected with *C. parvum* oocysts, and clinical signs as well as parasite load were assessed in the infected animals. Two days after the goats were infected with *C. parvum*, the feces became watery, with clumps and mucus, and changed color from brown to yellow. At 2 dpi, the average fecal consistency score increased from 0 to 3.6 in the infected group (Figure 1A). Diarrhea was associated with high dehydration scores that persisted for 20 dpi, with a brief period of normalization at 12 dpi (Figure 1B). Infection also produced hypothermia, with lower temperatures registered from 3 to 8 dpi (Figure 1C). The infected animals showed growth retardation during the acute illness (GLMM: infection, *F* = 186.9, *P* < 0.001; time, *F* = 164.5, *P* < 0.001; infection × time, *F* = 36.8, *P* < 0.001; animal variance = 0.27), with differences in average weight of 3.45 kg at 25 dpi compared to the control group (Figure 1D). Finally, high (80%) mortality (Mantel–Cox χ^2^-based test; *P* < 0.001) was observed in the group of infected animals (Figure 1E).

Infection was confirmed by IFA and qPCR in feces samples collected daily (Figure 1F). IFA revealed that oocyst shedding started at 4 dpi, peaked at 6 dpi, and ceased from 13 dpi (Figure 1F). These results were confirmed by qPCR (Figure 1F), with trends similar to the IFA results.

### Acute Infection of *C. parvum* Reduces the Diversity of Goat Kids’ Microbiota

**FIGURE 2:**
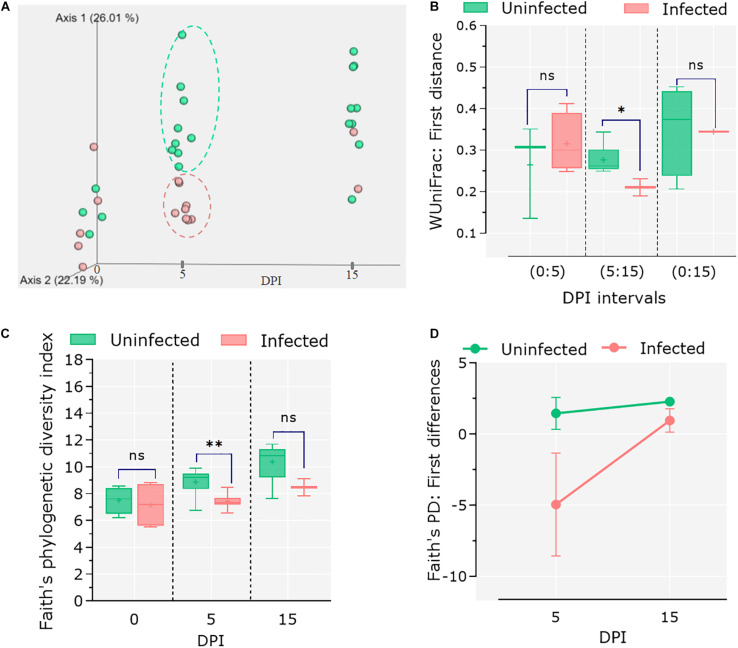
Differential microbial diversity in goat kid microbiota due to *Cryptosporidium parvum* infection. **(A)** Principal coordinate analysis (PCoA) of weighted UniFrac distances. We chose to represent the duration of infection on the third principal axis (0, 5, and 15 dpi). Each dot represents an individual microbiome (green = uninfected, red = infected); ellipsoids were arbitrarily drawn to highlight the difference between groups at 5 dpi. **(B)** Pairwise weighted UniFrac distance (degree of dissimilarity between an individual’s microbiota structure at two separate time points) indicates the distance (dissimilarity) between paired samples from uninfected and infected animals in relation to infection time (dpi). We tested whether these differences were significant at three time intervals (dpi): 0–5, 5–15, and 0–15. **(C)** Comparison of alpha diversity between uninfected and infected goat kids as measured by the “Faith” phylogenetic index (species richness) at genus level at 0, 5, and 15 dpi. **(D)** Pairwise difference comparisons addressing the changes in Faith phylogenetic index between paired samples in relation to infection time (i.e., species richness in each individual at 5 dpi in relation to itself at 0 dpi and microbiota differences as compared between infected and uninfected animals). Error bars represent standard deviation. Statistical significance was determined by Kruskal–Wallis test (n.s., ^∗^*P* < 0.05, ^∗∗^*P* < 0.01, ^∗∗∗^*P* < 0.001).

To assess the impact of *C. parvum* infection on the host gut microbiota, the feces of infected and control animals were collected at different time points (i.e., 0, 5, and 15 dpi), and 16S rRNA amplicon sequencing was performed. Sequencing data were used to analyze changes in the microbiome during the course of infection as well as comparing these changes to the healthy microbiota dynamics in the control group. Analysis of β-diversity revealed no differences in the microbiota composition between infected and uninfected animals at 0 dpi. However, significant differentiation was observed in the infected animals at 5 dpi, while at 15 dpi the microbiota β-diversity of the two animals who survived the *C. parvum* infection (*n* = 2) was similar to that of the control group (Figure 2A). Statistical comparisons using the Adonis test confirmed that both infection and time influenced the β-diversity of host microbiota (Adonis: infection, *P* = 0.01; time, *P* = 0.01). The longitudinal analysis based on weighted UniFrac distance (i.e., degree of dissimilarity between an individual’s microbiota community structure at two separate time points (Bokulich et al., 2018a) revealed differences in stability and/or evolution of the goat kid microbiota under the influence of *C. parvum* infection (Figure 2B). The microbiota of an infected animal at 5 dpi was significantly different to their compositions at 0 dpi than in uninfected animals. However, when compared in the interval from 5 to 15 dpi, the microbiota of infected animals showed less variability (Wilcoxon rank sum tests; *P* = 0.01), suggesting that the normal evolution of goat kid gut microbiota was hampered due to *C. parvum* infection and its associated acute illness.

On the other hand, species richness (α-diversity), assessed with Faith’s phylogenic diversity index, showed a steady increase from 0 to 15 dpi in the control group, while it did not change in the *C. parvum*-infected animals (Figure 2C). The differences between groups were only significant at 5 dpi (Kruskal–Wallis: *P* = 0.03). A longitudinal analysis showed that the microbiota of infected animals decreased in bacterial species (or ASVs) richness compared to its composition at 0 dpi, but the animals that survived the acute illness recovered the taxonomic richness of the microbiota when assessed at 5 and 15 dpi (Figure 2D).

### *C. parvum* Infection Impairs the Normal Development of the Gut Microbiota and Enhances the Abundance of Clostridiales

The taxonomic structure of gut microbiomes was measured in uninfected and infected kid goats with the purpose of identifying differences in abundance and taxonomic composition associated with *C. parvum* infection. An exploratory analysis using Gneiss test showed that the microbial communities were similar in feces from infected and uninfected animals at 0 dpi, while at 5 and 15 dpi, substantial differences were observed in the taxonomic composition between the groups (Supplementary Figure 1). Two trends were identified in the taxonomic differentiation induced by infection: (i) several taxa emerged in the uninfected animals at 5 and 15 dpi when compared with their initial compositions at 0 dpi, whereas these taxa did not increase in the infected animals, and (ii) in the infected animals (at 5 and 15 dpi), other taxa arose that were more abundant than in the uninfected animals.

**FIGURE 3:**
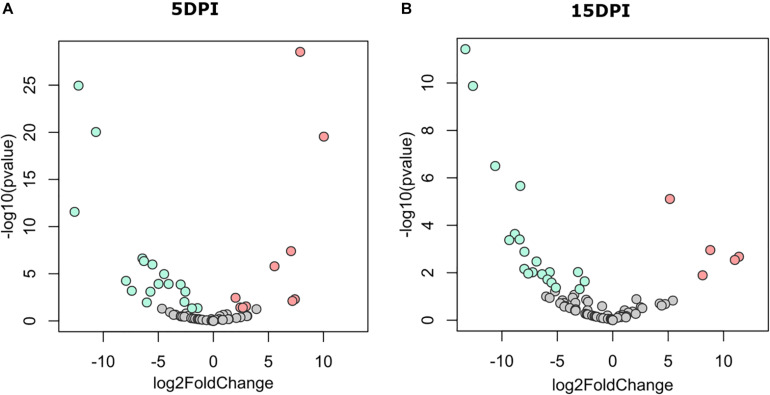
Differential abundant taxa between fecal microbiota of goat kids, from groups uninfected and infected with *Cryptosporidium parvum.* Volcano plot showing differential abundant taxa (green = uninfected, red = infected, gray = non-significant) identified by DESeq2 analysis. Taxa and ranked by log fold-change and the negative log-10 transform of the nominal *P*-value (*P* < 0.05). Comparisons were performed at **(A)** 5 dpi and **(B)** 15 dpi. Detailed information on taxa identity and differentiation is presented in Supplementary Table 1.

The discriminant analysis between infected and uninfected animals at 5 dpi revealed the identity of 10 or 17 bacterial genera that increased in the infected or the healthy animals, respectively (Figure 3A). Among the bacterial genera with higher fold-change ratios in favor of uninfected animals were *Dialister*, *Intestinibacter*, *Sellimonas*, *Anaerotruncus*, *Enterobacter*, *Mogibacterium*, and *Bifidobacterium*, whereas in the infected animals *Clostridioides*, *Lysinibacillus*, *Clostridium sensu stricto* 1, *Clostridium sensu stricto* 2, *Vagococcus*, *Sutterella*, *Proteus*, and *Tyzzerella* increased (Supplementary Table 1). Differences between groups remained similar at 15 dpi (Figure 3B), although three new genera from the family *Ruminococcaceae* [i.e., *Intestinimonas*, *Eubacterium* (coprostanoligenes group), and *Ruminococcaceae UCG-004*] arose in uninfected animals and were found at the top of the fold-change ranking, while other Clostridiales, including *Epulopiscium* and *Romboutsia*, were increased in the infected animals (Supplementary Table 1). These results suggest that *C. parvum* acute infection favors the colonization of some bacterial taxa over others, thus modulating gut microbiota composition in goats.

**FIGURE 4:**
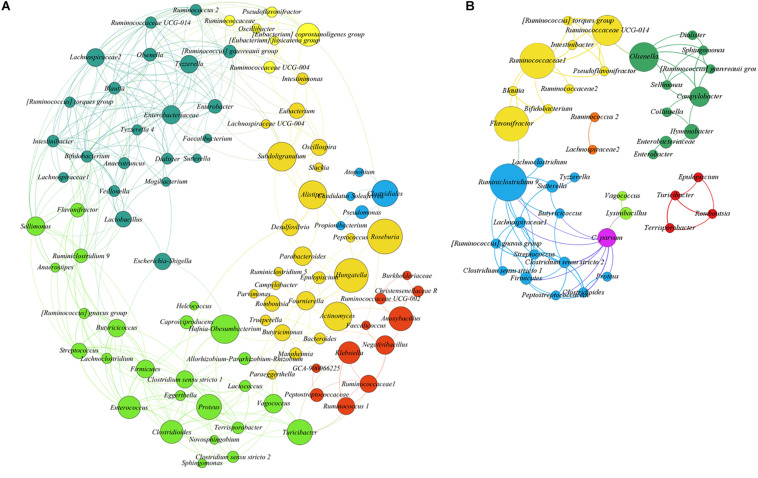
SparCC correlation networks observed between taxa, obtained from bacterial and archaeal 16S rRNA gene sequences. Nodes correspond to taxa at the genus level, and connecting edges indicate positive correlations larger than 0.50 (only nodes with at least one significant correlation are represented). **(A)** Co-occurrence network using all samples from uninfected goat kids. **(B)** Co-occurrence network using all samples from the infected animals, including *Cryptosporidium parvum* infection levels as a node (oocysts per gram of feces quantified by qPCR). Node colors are random, but those with the same color indicate taxa modules (communities) that co-occur more frequently than with other taxa. The circle size is proportional to the betweenness centrality of each taxon in the resulting network.

To test the hypothesis that acute *C. parvum* infection favors the colonization of a specific set of microbial taxa in goat microbiota, we measured the impact of parasitic load (OPG addressed by qPCR) on bacterial co-occurrence networks (Layeghifard et al., 2017). The co-occurrence networks revealed that the microbial communities in uninfected animals had more positive correlations between genera than controls (Supplementary Table 2). Also, the number of connected genera (nodes) (Figure 4A) in each module was higher in non-infected animals than in *C. parvum*-infected animals (Figure 4B). In addition, a network analysis confirmed that the parasitic load of *C. parvum* positively correlated (SparCC > 0.5) with the abundance of nine bacterial genera, including those identified in the differential taxonomic analysis as significantly more abundant in infected animals (Figure 3 and Supplementary Table 1), including *Lysinibacillus*, *Clostridioides*, *Clostridium sensu stricto* 1, *Clostridium sensu stricto* 2, *Vagococcus*, *Ruminiclostridium 9*, and *Proteus*.

### Microbiota Modulation by *C. parvum* Affects the Functional Profile of Goat Kid Microbiome

To assess the impact of microbial taxa modulation by *C. parvum* on the functional profiles of goat gut microbiomes, we performed pathway profiling based on predicted metagenomic functions using the bioinformatic tool PICRUSt2 (Douglas et al., 2020). A total of 387 pathways were identified in all the samples. We then explored variations between groups in a two-step analysis: (i) functional profiles were analyzed in both sample groups using Gneiss test and (ii) pairwise comparisons were made between infected and uninfected animals at 0, 5, and 15 dpi. Gneiss analysis revealed that the most significant differences in the functional profiles between groups were observed at 5 and 15 dpi. In addition, several of the identified pathways increased in uninfected animals at 5 and 15 dpi compared to 0 dpi, but this increase was not observed in the infected group (Supplementary Figure 2). These results suggest that the evolution and the development of the goat kid microbiome are associated with an increase in the abundance of some pathways and that normal microbiome development is interrupted by *C. parvum* infection.

**FIGURE 5:**
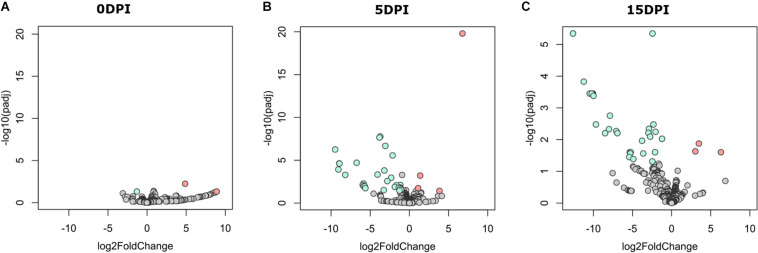
Differential functional profile of goat kid microbiome from uninfected and *Cryptosporidium parvum*-infected goats. Volcano plot showing differential abundant pathways between infected and uninfected animals detected by DESeq2 analysis at **(A)** 0 dpi, **(B)** 5 dpi, and **(C)** 15 dpi. The green and the red dots indicate pathways that display both large-magnitude log fold-changes and negative log-10 transform of the adjusted *P*-value (Benjamini–Hochberg false discovery rate method); the gray dots are not significant (*P*_*adj*_ < 0.05). Detailed information on pathway identity and differentiation is presented in Supplementary Table 3.

A pairwise comparison of functional profiles between groups at different time points revealed little significant difference between groups at 0 dpi (Figure 5A), where the pathways PWY-6545 (pyrimidine deoxyribonucleotide *de novo* biosynthesis III) and PWY-7431 [aromatic biogenic amine degradation of PWY-5531 (3,8-divinyl-chlorophyllide a biosynthesis II) and PWY-7159 (3,8-divinyl-chlorophyllide a biosynthesis III)] were significantly most abundant in uninfected and infected animals, respectively. However, several pathways were found to be more abundant in uninfected animals at 5 (Figure 5B) and 15 dpi (Figure 5C), respectively (Supplementary Table 3). Among the pathways with lower abundance in infected animals at 5 and 15 dpi were those related to the degradation of aromatic compounds (gallate and toluene) and the biosynthesis and the degradation of carbohydrates and amino acids. Pathways related to the biosynthesis of short-chain fatty acids (SCFA) likewise had a lower abundance in infected animals at both 5 and 15 dpi.

### Acute Infection by *C. parvum* Decreases the Relative Abundance of Pathways Involved in the Biosynthesis of SCFA

**FIGURE 6:**
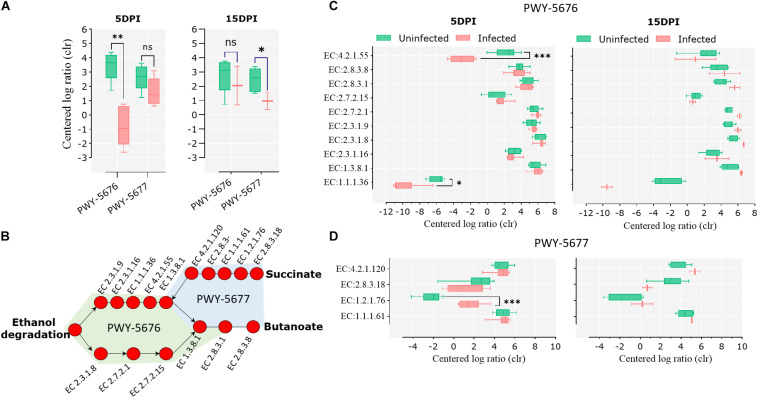
Short-chain fatty acid (SCFA) biosynthesis pathways in goat kid gut microbiomes affected by *Cryptosporidium parvum* infection. **(A)** Differential abundance of the SCFA pathways PWY-5676 (fermentation of acetyl-CoA to butanoate II) and PWY-5677 (fermentation of succinate to butanoate) between uninfected and infected animals at different infection time points (dpi). **(B)** SCFA pathway reconstruction using MetaCyc pathway maps, indicating all and Kyoto Encyclopedia of Genes and Genomes (KEGG) enzymes involved in such pathways. Differential abundance of KEGG enzymes from the pathways PWY-5676 **(C)** and PWY-5677 **(D)** at 5 and 15 dpi between uninfected and infected goat kids. Statistical significance was determined by Kruskal–Wallis test (^∗^*P* < 0.05, ^∗∗^*P* < 0.01, ^∗∗∗^*P* < 0.001).

Pathways involved in the fermentation of non-digestible carbohydrates to SCFA, specifically the fermentation of acetyl-CoA to butanoate II (PWY-5676) and the fermentation of succinate to butanoate (PWY-5677), were under-represented in the infected animals at 5 and 15 dpi, respectively (Figure 6A). A pathway reconstruction analysis revealed the presence of all the genes encoding for the enzymes involved in PWY-5676 and PWY-5677 in goat kid microbiomes (Figure 6B) and helped to identify the enzymes with significant changes at 5 and 15 dpi. The enzymes acetoacetyl-CoA reductase (EC 1.1.1.36) and 3-hydroxybutyryl-CoA dehydratase (EC 4.2.1.55), of PWY-5676, were relatively less abundant in infected animals at 5 dpi (Figure 6C), and the enzyme succinate-semialdehyde dehydrogenase (EC 1.2.1.76), of PWY-5677, was more abundant in infected animals at 5 dpi (Figure 6D). The enzymes propionate kinase (EC 2.7.2.15) and succinyl-CoA:acetate CoA-transferase (EC 2.8.3.18) showed a strong tendency to a lower abundance in infected animals at 15 dpi. However, the difference was not significant, likely due to only two available data points representing the surviving animals from the infected animal group.

### *C. parvum* Infection Decreases the Amount and the Abundance of Taxa Containing SCFA Biosynthesis Pathways in Goat Kid Microbiomes

**FIGURE 7:**
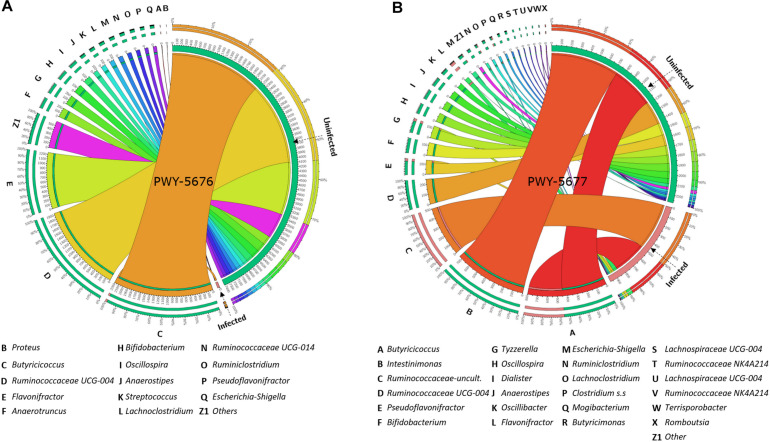
Taxa (16S) contribution to short-chain fatty acid (SCFA) biosynthesis pathways (PICRUSt2-predicted EC metagenome) affected by *Cryptosporidium parvum* infection in goat kid microbiomes. Chord diagrams showing the inter-relationships between taxa and SCFA pathways and these relationships compared between uninfected and infected goat kids for **(A)** PWY-5676 (fermentation of acetyl-CoA to butanoate II) at 5 dpi and **(B)** PWY-5677 (fermentation succinate to butanoate) at 15 dpi. Node segments along a circle represent taxa or the analyzed pathways in each group (infected and uninfected). The node size indicates the abundance (feature count) of contributing taxa and pathways. Arcs indicate the connections between pathways and taxa, which are represented proportionally by the size of each arc.

With the purpose of testing a possible association between the abundance of SCFA biosynthesis pathways and the taxonomic composition of goat kid microbiome, the amount and the abundance of bacterial genera with genomic contribution to PWY-5676 and PWY-5677 were quantified. The number of taxa contributing to both PWY-5676 and PWY-5677 was significantly lower in infected animals at 5 and 15 dpi, respectively, that is, at 5 dpi, PWY-5676 was provided by 36 bacterial genera in uninfected animals, while only *Butyricicoccus* contributed to PWY-5676 in infected animals. Although six other genera harbored the implicated genes, they were represented in low numbers (Figure 7A). At 15 dpi, 36 genera likewise contributed to PWY-5677 in uninfected animals, but only 11 harbored these genes in the microbiomes of infected animals (Figure 7B). In addition, the decreased number of taxa contributing to SCFA biosynthesis pathways was associated with *C. parvum* infection. The greatest effect on such taxa abundance was the decrease of *Butyricicoccus*, *Flavonifractor*, *Ruminococcaceae* UCG-004, *Intestinimonas*, *Bifidobacterium*, and *Oscillospira*, among others, in infected animals. These results suggest that microbiota modulation by *C. parvum* impacts the functional profiles of goat kid microbiomes and SCFA biosynthesis pathways in particular.

## Discussion

In this study, we tested the impact of *C. parvum* infection on the taxonomic and the functional profiles of goat gut microbiome using a clinically relevant model of cryptosporidiosis in goat kids. To this end, we performed high-throughput 16S rRNA gene sequencing analysis on genomic DNA collected from fecal samples at different time-points post-infection (0, 5, and 15 dpi) to assess the evolution of the gut microbiome in infected and uninfected animals. In addition, we predicted the metabolic profiles of microbiomes using the novel bioinformatics tool PICRUSt2, which enabled us to investigate the functional traits of the microbiome under disease pressure. PICRUSt2 is more accurate than other metagenome predictive algorithms as it uses all surveyed 16s ASVs (through sequence alignment) in a vast catalog of reference metagenomes (Douglas et al., 2020). The algorithm was updated with the new denoising methods (i.e., DADA2), thus enabling sequence analysis resolution down to the single-nucleotide level and enabling the differentiation of closely related organisms and more precise 16S sequence gene annotations (Douglas et al., 2020). Nevertheless, we are aware of the limitations of gene predictions. Hence, these results will be validated by shotgun metagenomics data and/or metatranscriptomic data in the future.

Our results showed that the gut microbiota diversity of goat kids was dramatically altered during early *C. parvum* infection (i.e., 5 dpi); however, with time (i.e., 15 dpi), the microbiomes reached levels of diversity and the microbial composition was similar to that of the control group. Although this study is limited by the co-housing of animals, which can influence the microbiota, and the unequal group size at 15 dpi, our results could hypothetically demonstrate that although goat kids are highly sensitive to acute *C. parvum* infection (high mortality and growth retardation), their microbiota tends to return to a normal state in animals that survived the acute infection. A similar trend was observed in natural *Cryptosporidium* infections in Coquerel’s sifakas (McKenney et al., 2017). Infection decreased the microbial diversity by depleting several specific taxa, after which the gut microbiome gradually recovered its original stable state (McKenney et al., 2017). This suggests that gut microbiomes have simultaneously low resistance to disruption but also high resilience (the rate of recovery after a disturbance) to *C. parvum* infection, which could be considered as a generator of disruption and dysbiosis in host gut microbiota (Shade et al., 2012). In addition, *Cryptosporidium* infections in Coquerel’s sifakas were distinguished by six biomarkers (McKenney et al., 2017), two of which, *Enterococcus* and Clostridiales, were also found to be increased upon *C. parvum* infection in goat kids.

Our results indicate that *C. parvum* infection-induced dysbiosis interferes with the normal development of the microbiota because, despite recovering its diversity after the disturbance, the butyrate biosynthesis pathways remained altered, even at 15 dpi, when parasite shedding in feces was undetectable. Our results therefore suggest that the normal development of goat kid microbiota sees an increase in the abundance, and thus perhaps the importance, of bacteria that produce butyrate, which likely increases gut tolerance to further bacterial colonization because butyrate is an anti-inflammatory molecule (Chang et al., 2014; Singh et al., 2014). The fact that *C. parvum* dramatically decreases the number and the relative abundance of butyrate-producing bacteria suggests that the intestinal inflammation associated with *C. parvum* infection in the gut (Kirkpatrick et al., 2002) may be related, at least in part, with decreased intestinal butyrate levels concomitant to reduced numbers of butyrate-producing bacteria. Infection with the helminth *H. contortus* was also found to modulate gut butyrate biosynthesis by altering the abundance of butyrate-producing bacteria (Li et al., 2016). Butyrate-producing bacteria such as *Ruminococcus*, *Clostridium* XIVa, and members of the *Lachnospiraceae* family were also decreased in ponies susceptible to gastrointestinal strongyles infection, but not in ponies resistant to this disease (Clark et al., 2018).

## Conclusion

In this study, we established a model of cryptosporidiosis in goat kids and evaluated the response of gut microbiome to *C. parvum* infection. Acute infection by *C. parvum* modulated the taxonomic and the functional profiles of the host–microbiome. Remarkably, pathways involved in the biosynthesis of the short-chain fatty acid butyrate were affected at both 5 and 15 dpi. This suggests that a decrease in butyrate from bacteria may be an indicator of *C. parvum* infection that contributes to the intestinal inflammation associated with cryptosporidiosis. These results reveal novel insights into host–microbiome–parasite interactions that can be used to develop novel methods for cryptosporidiosis control in livestock.

## Author’s Note

The research published in this manuscript was performed by MM while doing his Ph.D. under the supervision of KA.

## Data Availability Statement

All datasets presented in this study are included in the article/Supplementary Material.

## Ethics Statement

All experiments were conducted following the guidelines of the Directive 2010/63/UE of the European Parliament and the Council, in the facilities of the Plate-Forme d’Infectiologie Expérimentale: PFIE, UE-1277, INRAE Centre Val de Loire, Nouzilly, France. All experimental procedures were approved by the Loire Valley ethical review board (CEEA VdL, committee number 19). Enrichment material has been made available for goats to maintain animal welfare.

## Author Contributions

MM, BP, CJ, TP, and KA conceived the study. MM and AC performed the experiments and acquired the data. MM, AC-C, and DO drafted the first version of the manuscript and analyzed the metabolic profiles and performed the pathway analysis. DO analyzed the 16S data set and obtained the results. All the authors revised and accepted the final version of the manuscript.

## Conflict of Interest

MM and CJ are employees of the company Phileo by Lesaffre. The research published in this manuscript was performed by MM while doing his Ph.D. under the supervision of KA. The remaining authors declare that the research was conducted in the absence of any commercial or financial relationships that could be construed as a potential conflict of interest.

## Footnotes

1 www.microbiome-standards.org

2 www.genoscreen.com

3 https://www.genoscreen.fr/fr/

4 www.ncbi.nlm.nih.gov/sra

## Supplementary Material

Supplementary Figure 1 Identification of differential taxa (genera) in the taxonomic profile of goat kids affected by *Cryptosporidium parvum* infection. Dendrogram heat map resulting from Gneiss analysis; taxa were clustered according to balances (ratios of taxa or groups of taxa) based on their abundance (centered log ratio). Nine balances (y0, y1,…, y9) were calculated based on hierarchical clusters on taxa abundance. Clusters I, III, and IV encompass the main taxa that differentiate between infected and uninfected goat kids at 5 and 15 dpi and compared to the initial situation at 0 dpi.

Supplementary Figure 2 Identification of differential pathways across all the samples. Dendrogram heat map resulting from Gneiss analysis; pathways were clustered according to balances (ratios of pathways or groups of pathways) based on their abundance (centered log ratio). Nine balances (y0, y1, …, y9) were calculated based on hierarchical clusters on pathway abundance (centered log ratio-transformed).

Supplementary Table 1 List of differential taxa identified by the DESeq2 method between the gut microbiota of infected and uninfected goat kids at 5 and 15 dpi (days post-infection). Differential taxa are ranked based on the log fold-changes and negative log-10 transform of *P*-value.

Supplementary Table 2 Topological features of the co-occurrence networks on fecal microbiomes from goat kids uninfected or infected with *Cryptosporidium parvum.*

Supplementary Table 3 List of differential pathways identified by the DESeq2 method between the gut microbiota of infected and uninfected goat kids at 0, 5, and 15 dpi (days post-infection). Differential pathways are ranked based on the log fold-changes and negative log-10 transform of *P*_*adj*_-value (Benjamini–Hochberg false discovery rate method).
