# Supplementary figures and images for: Cryptosporidium parvum Infection Depletes Butyrate Producer Bacteria in Goat Kid Microbiome
Source: Front Microbiol. 2020 Oct 16;11:548737. doi: 10.3389/fmicb.2020.548737 (PMC7596689; doi:10.3389/fmicb.2020.548737)

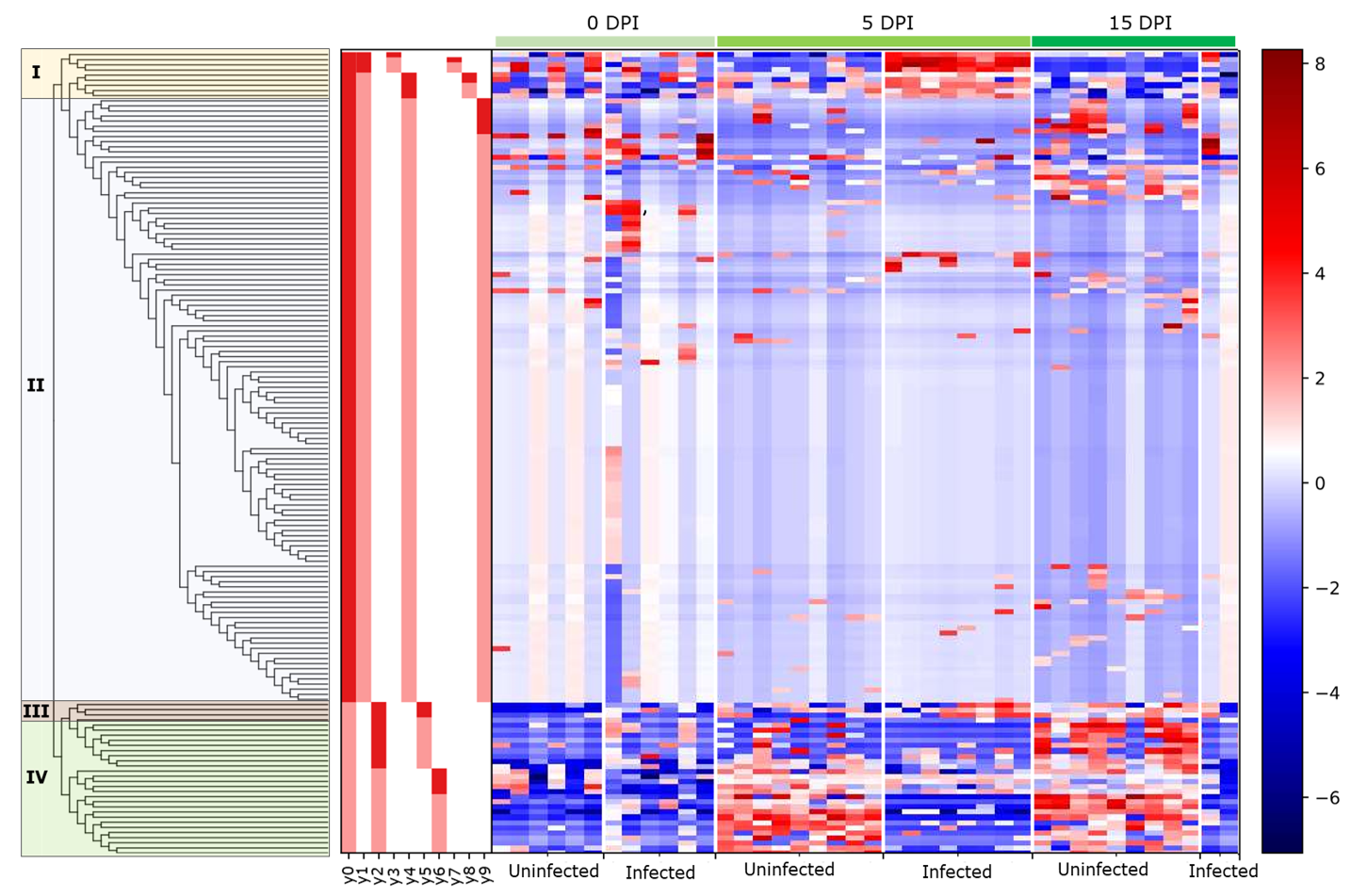

Supplement: Supplementary Figure 1 — Identification of differential taxa (genera) in the taxonomic profile of goat kids affected by Cryptosporidium parvum infection. Dendrogram heat map resulting from Gneiss analysis; taxa were clustered according to balances (ratios of taxa or groups of taxa) based on their abundance (centered log ratio). Nine balances (y0, y1,…, y9) were calculated based on hierarchical clusters on taxa abundance. Clusters I, III, and IV encompass the main taxa that differentiate between infected and uninfected goat kids at 5 and 15 dpi and compared to the initial situation at 0 dpi. [file Image_1.TIF]

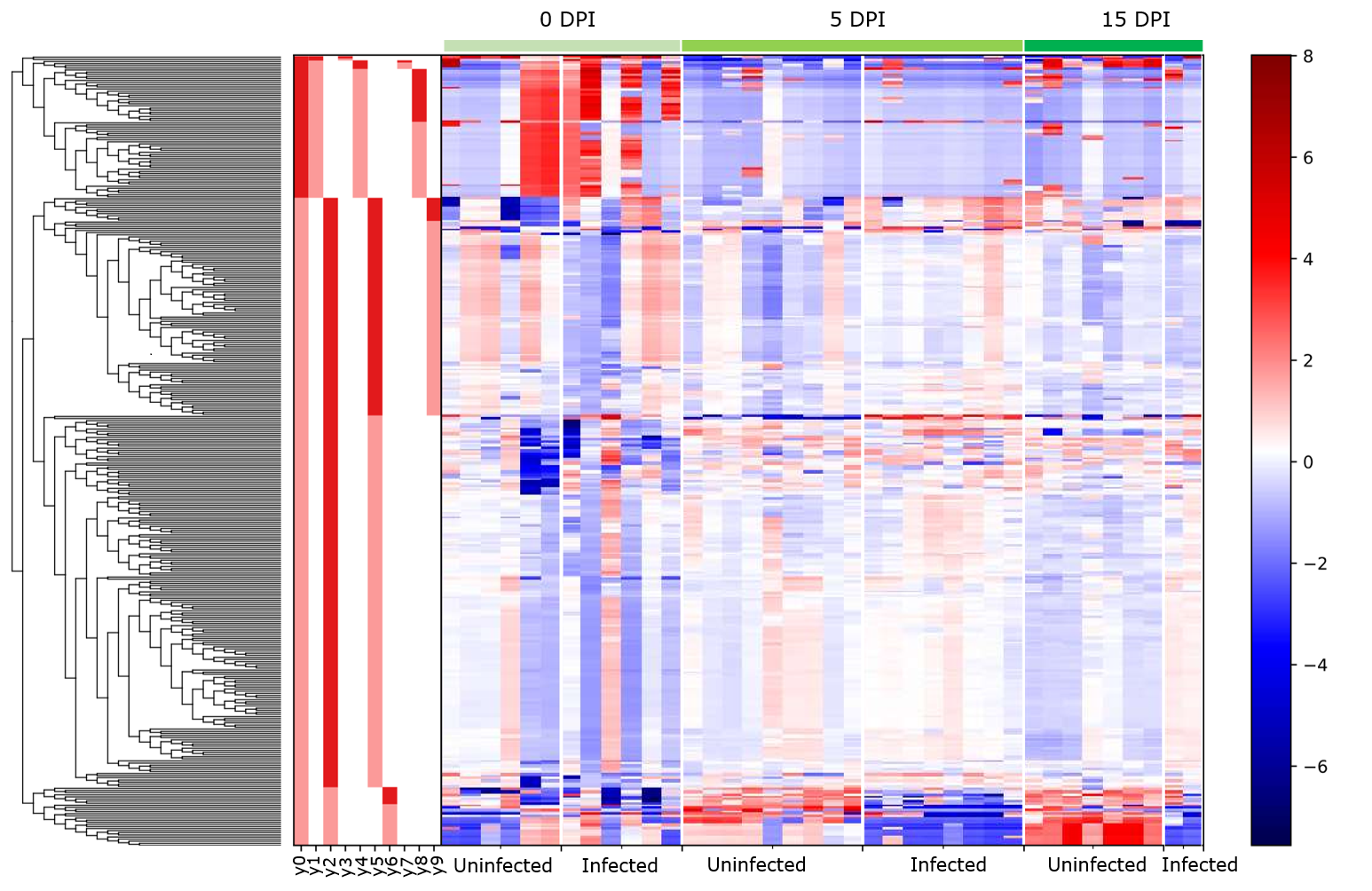

Supplement: Supplementary Figure 2 — Identification of differential pathways across all the samples. Dendrogram heat map resulting from Gneiss analysis; pathways were clustered according to balances (ratios of pathways or groups of pathways) based on their abundance (centered log ratio). Nine balances (y0, y1, …, y9) were calculated based on hierarchical clusters on pathway abundance (centered log ratio-transformed). [file Image_2.TIF]
