# Supplementary material for: Cryptosporidium parvum Infection Depletes Butyrate Producer Bacteria in Goat Kid Microbiome
Source: Front Microbiol. 2020 Oct 16;11:548737. doi: 10.3389/fmicb.2020.548737 (PMC7596689; doi:10.3389/fmicb.2020.548737)
Supplement: Supplementary Table 3 — List of differential pathways identified by the DESeq2 method between the gut microbiota of infected and uninfected goat kids at 0, 5, and 15 dpi (days post-infection). Differential pathways are ranked based on the log fold-changes and negative log-10 transform of Padj-value (Benjamini–Hochberg false discovery rate method). [file Table_3.DOCX]

**Supplementary Table 1.** Topological features of the co-occurrence networks on fecal microbiome from goat kids uninfected and infected with *C. parvum*

| Topological features | Uninfected | | Infected |
| --- | --- | --- | --- |
| Nodes^a^ | 95 | 42 | |
| Modules^b^ | 6 | 6 | |
| Network diameter^c^ | 9 | 11 | |
| Average degree^d^ | 7.43 | 3.90 | |
| Weighted degree^e^ | 3.13 | 2.40 | |
| Average clustering coefficient^f^ | 0.47 | 0.6 | |

^a^ Taxa with at least a significant (*P* < 0.01) and positive (SparCC > 0.5) correlation;

^b^Modules are formed by a group of nodes densely connected. A higher number of modules means for a higher number of functions that do not co-occur frequently and slightly interact with other functions;

^c^ The longest distance between nodes in the network. The longest the distance the less robust is the network;

^d^ The average number of connections per node in the network, that is, the node connectivity;

^e^Weighted degree is the sum of the weights of all links attached to a node.

^f^ The average clustering coefficient indicates how nodes are embedded in the network. High values indicate the presence of modules with well-connected nodes but isolated from other modules.
